# Supplementary material for: International Perspectives of Extended Genetic Sequencing When Used as Part of Newborn Screening to Identify Cystic Fibrosis
Source: Int J Neonatal Screen. 2024 Apr 8;10(2):31. doi: 10.3390/ijns10020031 (PMC11036278; doi:10.3390/ijns10020031)
Supplement: Supplementary file 1 [file IJNS-10-00031-s001.zip › IJNS-2876460-supplementary.pdf]

Questionnaire: Health Professionals Views of extended genetic sequencing for CF

Start of Block: Information and Consent

Q1 Thank you for taking the time to read the information sheet.

We are interested to understand the views of professionals with respect to newborn bloodspot screening (NBS) for cystic fibrosis and the potential for using more extensive gene sequencing in the protocol.

Please tick the relevant box below to proceed.
I confirm that I have read the information sheet.

I have had the opportunity to consider the information, ask questions and have had these answered satisfactorily.

I understand that my participation is voluntary and that I am free to withdraw at any time without giving any reason and without my legal rights being affected.

I understand that the information collected will be used to support other research in the future, and may be shared anonymously with other researchers.

I agree to complete the questionnaire.

○ Yes (1)

○ No (2)

Skip To: End of Survey If Thank you for taking the time to read the information sheet for the study titled: Exploring perce... = No

End of Block: Information and Consent

Start of Block: Section 1: Demographic details

This section collects demographic details about you

Q1 In which country do you currently work?

- ○ US
- ○ Canada
- ○ Australia
- ○ New Zealand
- ○ United Kingdon
- ○ Austria
- ○ Belgium
- ○ Bulgaria
- ○ Croatia
- ○ Cyprus
- ○ Czech Republic
- ○ Denmark
- ○ Estonia
- ○ Finland
- ○ France
- ○ Germany
- ○ Greece
- ○ Hungary
- ○ Italy
- ○ Latvia

○ Lithuania

○ Luxembourg

○ Malta

○ Netherlands

○ Poland

○ Portugal

○ Romania

○ Slovakia

○ Slovenia

○ Spain

○ Sweden

○ Switzerland

○ Ireland

○ Turkey

○ Other, please specify ____

Q2 Please specify the state or region you work within in this country.

____

Q3 Which best describes your professional role with children/families with experience of cystic fibrosis (affected, carrier, CFSPID designation)?

- ◯ CF Consultant
- ◯ Paediatrician
- ◯ Transplantation surgeon
- ◯ CF Clinical Nurse Specialist
- ◯ Screening Nurse
- ◯ Nurse, other ____
- ◯ Laboratory staff
- ◯ Genetic Counsellor
- ◯ Social worker
- ◯ Dietician
- ◯ Physiotherapist
- ◯ Centre Director
- ◯ Professor / Associate Professor
- ◯ Research Scientist
- ◯ Newborn Screening Coordinator
- ◯ Support worker / advocate
- ◯ Other, please state ____

Q4 What are your professional qualifications?

- ▢ MD / MBChB / MBBS or equivalent (1)
- ▢ PhD (2)
- ▢ MSc (3)
- ▢ BSc/BA (4)
- ▢ RN (5)
- ▢ Other, please state (8) ____

Q5 How many years have you worked with children/adults/families with experience of cystic fibrosis (affected, carrier, CFSPID designation)?

____

End of Block: Section 1: Demographic details

Start of Block: Section 2: Your views of incorporating NGS into NBS for CF

This section focuses on your views of incorporating extended genetic sequencing into newborn bloodspot screening (referred to from now on as 'screening') for cystic fibrosis (CF).

What will incorporating extended genetic sequencing (EGS) into CF screening mean?
Incorporating EGS, will mean that the analysis is more comprehensive in terms of the number of gene mutations that are screened; all babies with an elevated IRT will be screened for more than 300

mutations compared with the 4 mutation panel, followed by (up to) 50 mutation panel currently used. Replacing these more limited panels seeks to achieve three main goals:

Avoiding reporting probable carriers (currently around 200 per annum)

Reducing the number of repeat IRT heel prick tests (currently around 300 per annum)

Ensuring that the mutations detected more adequately reflect the ethnic diversity in the population.

What is the current screening protocol?
The current UK screening protocol for CF is presented here to provide context for the scenarios described below.

Currently, screening results in babies being reported as:

1. CF not suspected - no further action required

2. Probable CF carrier (one CF causing gene but not affected by CF) - the family may be offered cascade screening (when the carriers' relatives are screened in a cascade fashion) and are offered advice to seek help if the child becomes unwell showing features associated with CF.

3. CF suspected - further confirmatory testing is required.

When a baby is reported as 'CF suspected' a clinical referral is made to undertake confirmatory testing. This may then result in a baby being classified as:

a. Normal (i.e. a false positive screening test result, the baby is then discharged)

b. CF confirmed (appropriate on-going treatment is arranged)

c. CF screen positive, inconclusive diagnosis (CFSPID) - these babies have a positive initial screening result, but further diagnostic testing is inconclusive.

The majority of these children remain well, while some may convert to a CF diagnosis at a later stage, and others may develop CFTR-related disorders. Consequently, identification of babies with the CFSPID designation can lead to uncertainty for both families and healthcare professionals as management of these infants continues to evolve.

What did the EGS analysis trial tell us?
During the EGS analysis trial, involving 70,000 babies, variants of varying clinical consequence were given a score = 1 per allele, and those with clearly pathogenic mutations = 2 per allele.

It was found that: Referring babies with a combined **score of 3** (one pathogenic mutation combined with one mutation of varying clinical consequence), would: 1. Result in an increase (from 25 per annum to 80 per annum in the UK) in the designation of infants with 'Cystic Fibrosis Screen Positive – Inconclusive Diagnosis' (CFSPID) 2. Be likely to help avoid a small number of missed CF cases when

compared with restricting reporting to those with a score of 4.
Referring babies with a combined **score of 4** (or two clearly pathogenic mutations) would: 1. Result in a reduction (from 25 per annum to 5 per annum in the UK) in the designation of infants with ‘Cystic Fibrosis Screen Positive – Inconclusive Diagnosis’ (CFSPID) 2. Create a possible chance (less than 10 per annum) of those with true CF being missed at screening - the majority of these babies will be diagnosed clinically by the age of two years

---

Q6 Given the information above, how important do you think it is to ensure babies with **true CF** are **not missed** (up to 10 babies per annum).

- ○ Very important
- ○ Moderately important
- ○ Neutral
- ○ Low importance
- ○ Not at all important

Q7 Please explain your reasoning below?

____

____

____

____

____

Q8 Given the information above, how important do you think it is to **reduce** the number of babies reported with a **CFSPID designation** (from 25 per annum to 5 per annum), by using a specific approach when compared with increasing this (from 25pa to 80pa) by using a sensitive apporoach?

- Very important
- Moderately important
- Neutral
- Low importance
- Not at all important

Q9 Please explain your reasoning below?

____

____

____

____

____

Q10 Overall, on the basis of the information provided, how important would it be to use a sensitive approach?

- Very important
- Moderately important
- Neutral
- Low importance
- Not at all important

Q11 Please explain your reasoning below?

____

____

____

____

____

Q12 Overall, on the basis of the information provided, how important would it be to use a specific approach?

- ○ Very important
- ○ Moderately important
- ○ Neutral
- ○ Low importance
- ○ Not at all important

Q13 Please explain your reasoning below?

____

____

____

____

____

Page Break

This section will involve ranking the possible outcomes mentioned in the previous section in terms of their importance.

Q14 Please could you indicate, using the scales below, how important you feel the following statements are (10= very important):

	0 1 2 3 4 5 6 7 8 9 10
Avoiding missing a baby with a true diagnosis ()	
Avoiding the identification of babies with a designation of cystic fibrosis screen positive, inconclusive diagnosis (CFSPID) ()	
Avoiding reporting probable carriers (currently around 200 per annum) ()	
Reducing the number of repeat IRT heel prick tests (currently around 300 per annum) ()	
Ensuring that the mutations detected more adequately reflect the ethnic diversity in the population ()	

End of Block: Section 2: Your views of incorporating NGS into NBS for CF

Start of Block: Supplemental questions

This section contains supplemental questions about the proposed use of EGS for CF screening that we would value your views on.

Page Break

Q15 How important do you think it is to **reduce or avoid** the number of babies being reported as '**probable CF carriers**' (those with one CF causing gene but may not be affected by CF) - currently 200 per annum?

- ○ Very important
- ○ Moderately important
- ○ Neutral
- ○ Low importance
- ○ Not at all important

Q16 Please explain your reasoning below?

____

____

____

____

____

Q17 Are you a health professional with experience of caring for children with CF and their families?

- ○ Yes
- ○ No

End of Block: Supplemental questions

Start of Block: Additional questions for health professionals

Display This Question:

If Are you a health professional with experience of caring for children with CF and their families? = Yes

Q18 In your experience, does a CFSPID designation impact on the family in any way?

- ◯ Yes, please describe how ________________
- ◯ Sometimes, please describe how ________________
- ◯ No

Display This Question:

If Are you a health professional with experience of caring for children with CF and their families? = Yes

Q19 In your experience, how frequently are children with a designation of CFSPID and their families reviewed by a clinical team?

- ◯ Annually
- ◯ 6 monthly
- ◯ Other, please specify ________________

Display This Question:

If Are you a health professional with experience of caring for children with CF and their families? = Yes

Q20 In your experience, are children with a CFSPID designation started on standard CF care pathways?

- ◯ Yes
- ◯ Sometimes, please state what would influence this decision ________________
- ◯ No

Display This Question:

If Are you a health professional with experience of caring for children with CF and their families? = Yes

Q21 Does this include CFTR modulator therapies?

- Yes
- Sometimes, please state what would influence this decision ____
- No

Display This Question:

If Are you a health professional with experience of caring for children with CF and their families? = Yes

Q22 What impact, if any, do you believe a delayed diagnosis of CF could have on the child both in the short term and in relation to longer term consequences on clinical outcomes?

____

____

____

____

____

Display This Question:

If Are you a health professional with experience of caring for children with CF and their families? = Yes

Q23 What impact, if any, do you believe a delayed diagnosis could have on the child's family?

____

____

____

____

____

End of Block: Additional questions for health professionals
